# Relationship between toxoplasmosis and obsessive compulsive disorder: A systematic review and meta-analysis

**DOI:** 10.1371/journal.pntd.0007306

**Published:** 2019-04-10

**Authors:** Tooran Nayeri Chegeni, Shahabeddin Sarvi, Afsaneh Amouei, Mahmood Moosazadeh, Zahra Hosseininejad, Sargis A. Aghayan, Ahmad Daryani

**Affiliations:** 1 Toxoplasmosis Research Center, Mazandaran University of Medical Sciences, Sari, Iran; 2 Department of Parasitology, School of Medicine, Mazandaran University of Medical Sciences, Sari, Iran; 3 Student Research Committee, Mazandaran University of Medical Sciences, Sari, Iran; 4 Health Sciences Research Center, Addiction Institute, Mazandaran University of Medical Sciences, Sari, Iran; 5 Laboratory of Zoology, Research Institute of Biology, Yerevan State University, Yerevan, Armenia; Universidade do Estado do Rio de Janeiro, BRAZIL

## Abstract

**Background:**

A few studies investigated the relationship between toxoplasmosis and mental disorders, such as obsessive compulsive disorder (OCD). However, the specific nature of the association between *Toxoplasma gondii* (*T*. *gondii*) infection and OCD is not yet clear. The aim of this study was to collect information on the relationship between OCD and toxoplasmosis and assess whether patients with toxoplasmosis are prone to OCD.

**Methods:**

For the purpose of this study, 6 major electronic databases and the Internet search engine Google Scholar were searched for the published articles up to July 30^th^, 2018 with no restriction of language. The inverse variance method and the random effect model were used to combine the data. The values of odds ratio (OR) were estimated at 95% confidence interval (CI).

**Results:**

A total of 9 case-control and 3 cross-sectional studies were included in our systematic review. However, 11 of these 12 articles were entered into the meta-analysis containing 9873 participants, out of whom 389 were with OCD (25.96% positive for toxoplasmosis) and 9484 were without OCD (17.12% positive for toxoplasmosis). The estimation of the random effect model indicated a significant common OR of 1.96 [95% CI: 1.32–2.90].

**Conclusion:**

This systematic review and meta-analysis revealed that toxoplasmosis could be as an associated factor for OCD (OR = 1.96). However, further prospective investigations are highly recommended to illuminate the underlying pathophysiological mechanisms of *T*. *gondii* infection in OCD and to better investigate the relationship between OCD and *T*. *gondii* infection.

## Author summary

*Toxoplasma gondii* (*T*. *gondii*) is an obligate neurotropic parasite that infected about 25–30% of the total human population in the developed and developing countries. The obsessive compulsive disorder (OCD) is a psychiatric disease that affects the income and quality of life. Some studies confirmed an association between infectious agents as the associated or protective factors specifying the development of psychiatry diseases. Among various pathogens associated with psychological disorders, most of the attention is on *T*. *gondii*, which has a life-long asymptomatic latent phase after a short acute stage in healthy individuals. The detrimental effect of *T*. *gondii* on immunocompromised people and pregnant women is an important concern for public health. The correlation between toxoplasmosis and OCD is still relatively understudied with a paucity of documented findings. The previous meta-analysis reviewed only two studies and reported a 3.4-fold greater chance of OCD. The results of our study presented stronger evidence of a positive relationship between toxoplasmosis and OCD. Eventually, our research team hopes to present an overview of what is known and encourage more intensive research to determine the real impact of this parasite on the occurrence of OCD that may contribute to the prevention of OCD worldwide.

## Introduction

The *T*. *gondii* is a neurotropic apicomplexan protozoan that infects one-third of the world’s human population by affecting some tissues, including brain, eyes, and testes in warm-blooded mammals [1]. Infection with this parasite is due to the consumption of raw or undercooked meat containing tissue cysts or consumption of food or drinking water contaminated with oocysts shed by cats. Moreover, organ transplantation, blood transfusion, and vertical transmission during pregnancy from mother to fetus are other causes of *T*. *gondii* transmission [2]. The *T*. *gondii* infection is generally asymptomatic in immunocompetent individuals. However, immunocompromised patients may experience severe clinical complications, such as chorioretinitis, encephalitis, and pneumonitis. Toxoplasmosis also leads to psychotic symptoms and changes in the personality of individuals [3]. The *T*. *gondii* has a specific tropism for brain tissue, where tachyzoites can invade to microglia, astrocytes, and neurons and create cysts in these cells. The considerable production of neurotransmitters, such as dopamine by *T*. *gondii*, induces the increased production of bradyzoites and destruction of cyst walls that may be responsible for behavioral changes [4,5].

Recently published systematic review and meta-analysis studies have examined the relationship between *T*. *gondii* infection and various psychiatric disorders; such as bipolar disorder [3,6], schizophrenia [6,7], epilepsy [8], and depression [6,9]. The results of these studies showed that toxoplasmosis is an associated factor for bipolar disorder, schizophrenia, epilepsy, but not for depression.

The OCD is a common, chronic, and debilitating psychiatric condition that affects about 3% of the general population [10,11]. This disorder is identified by unwanted and recurrent thoughts, which cause marked distress. Individuals with OCD are struggling to reduce their anxiety by mental acts and repetitive behaviors [12]. According to the World Health Organization, OCD is one of the top ten disorders which affect people’s income and quality of life although it has the least effect [13].

Some of the available data indicate the possibility of an association between toxoplasmosis and OCD [14,15] although there are some contradictory results [16]. Therefore, the main purpose of this systematic review and meta-analysis was to evaluate the relationship between *T*. *gondii* and OCD.

## Methods

### Design and protocol registration

This study was designed according to the Preferred Reporting Items for Systematic Reviews and Meta-Analysis (PRISMA) guidelines [17]. The protocol was registered in the PROSPERO with the registration number of CRD42018106354 [18].

### Search strategy

To identify the published studies on the association between toxoplasmosis and OCD, the researchers performed a systematic search in 6 databases, namely PubMed, Scopus, ScienceDirect, Web of Science, EMBASE, ProQuest, and the Internet search engine Google Scholar. This systematic review was conducted through gathering the articles published up to July 30^th^, 2018 with no restriction of language. The search process was accomplished using the following keywords “*Toxoplasma*” OR “toxoplasmosis” AND “Obsessive-Compulsive Disorder” OR “OCD”.

### Inclusion and exclusion criteria

The inclusion criteria included: (1) studies published until July 30^th^, 2018, (2) case-control and cross-sectional studies about the relationship between toxoplasmosis and OCD, (3) original research papers, (4) studies with available full texts, and (5) studies with information on the exact total sample size and positive samples in the case and control groups. The exclusion criteria were: (1) studies with no exact information about the sample size in the case and control groups, (2) review articles, and (3) non-human studies.

### Study selection and data extraction

**Fig 1:**
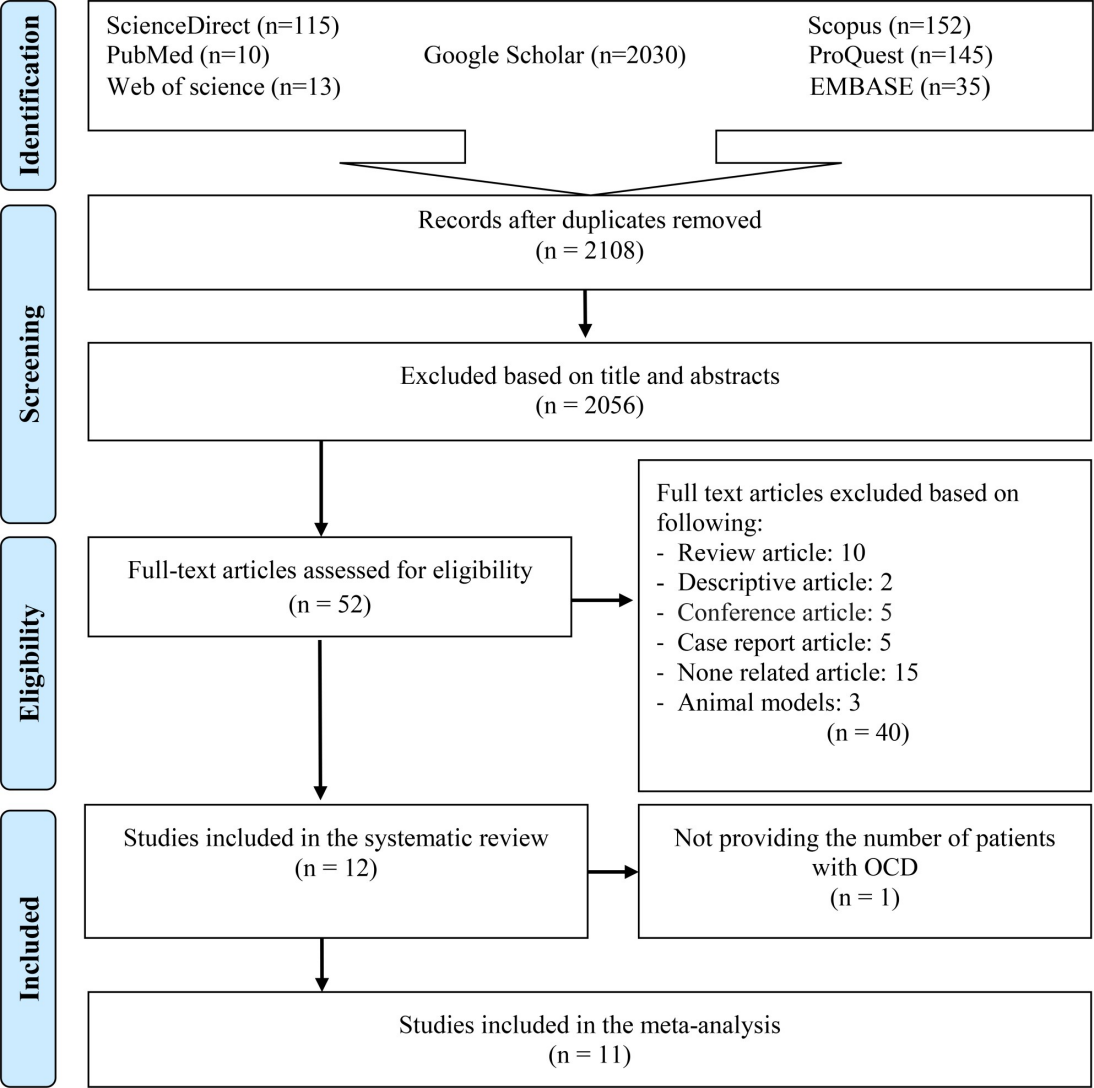
Flow diagram of the study design process.

All the retrieved articles from the search strategy were imported to EndNote (version X7). After the removal of duplicated papers, the titles and abstracts were independently reviewed by two researchers. In the next step, eligible articles were selected for full-text download (Fig 1). Data from relevant studies were extracted into a Microsoft Excel datasheet. The extracted variables included the name of first author, year of publication, location of the study, diagnostic method, OR, number of seropositive cases and control, as well as the age and gender of the participants in the case and control groups. The researchers of the current study were very careful about extracting the correct information. In this regard, the authors of the three selected articles were contacted for more detailed information [19–21].

### Quality assessment

Two researchers independently assessed the quality of the included papers using standard strengthening the Reporting of Observational Studies in Epidemiology checklist (STROBE). This scale includes 22 items that are related to the title, abstract, introduction, methods, results, and discussion sections of the articles. This checklist included items assessing objectives, different components of the methodology (e.g., study design, study size, study population, bias, statistical methods), key results, limitations, generalizability, and funding of the studies. The assigned scores were within the range of 0–44. Based on the STROBE checklist assessment, articles were categorized into 3 groups (low quality: less than 15.5, moderate quality: 15.5–29.5, and high quality: 30.0–44.0). The S1 Checklist indicates the quality of the included studies [22].

### Statistical analysis

The data entered into Microsoft Excel were exported to Stata version 14 (Stata Corp, College Station, TX, USA) for the analysis [23]. The common OR were estimated using inverse variance and random-effects model for each included study. Furthermore, the heterogeneity index was determined using Cochran’s Q and I squared statistics. I squared values less than 25%, 25–50%, and greater than 50% were defined as low, moderate, and high heterogeneity, respectively [23]. The publication bias was examined by the Egger test. A sensitivity analysis was performed using Stata version 14 (Stata Corp, College Station, TX, USA) to identify the possible effect of each study on the overall results by removing each study.

## Results

Out of 2500 identified articles, 392 articles were excluded due to the duplication, and 2056 articles were also eliminated on the basis of their titles and abstracts. After reading the full text of the articles, 12 papers were included in our systematic review [14–16,19–21,24–29]. Eventually, 11 of these 12 articles [14–16,19–21,24,25,27–29] were entered into this meta-analysis with respect to the inclusion/exclusion criteria (Fig 1). One of the papers was excluded due to the lack of detailed information about the number of patients with OCD [26]. Information and characteristics about the investigated publications are presented in Table 1 and Table 2.

**Table 1:** Description of the studies included looking for an association between toxoplasmosis and obsessive compulsive disorder.

No	First author	Publication year	Place of study	Type of study	Method	Test	Results	Age (years ± SD)	Sex (N)
1	Alvarado-Esquivel C [28]	2006	Mexico	Case control	ELISA	IgG IgM	Not significant	P: ≥16 C: 16–54	P: (-) C: (F:55, M:125)
2	Miman O [14]	2010	Turkey	Case control	ELISA IFA	IgG IgM	Significant	P: 18–70 C: 18–70	P: (F:25, M:17) C: (F:62, M:38)
3	Xiao Y [27]	2010	China	Case control	ELISA	IgG	Not significant	P: 15–65 C: 15–65	P: (-) C: (F:1315,M:1319)
4	Cong W [29]	2015	China	Case control	ELISA	IgG IgM	Not significant	P: 16–91 C: 16–91	P: (-) C: (F:238, M:207)
5	Memik NÇ [16]	2015	Turkey	Case control	ELISA	IgG IgM	Not significant	P: 11.84±3.19 C: 12.97±2.84	P: (F:23, M:19) C: (F:20, M:25)
6	Zaki WM [24]	2016	Saudi Arabia	Case control	ELISA	IgG IgM	Not significant	P: 19–67 C: 17–64	P: (-) C: (F:68, M:94)
7	Flegr J [19]	2016	Czech Republic	Case control	CFT ELISA	IgG IgM	Not significant	P:M: 34.0±10.5 F: 36.5±12.3 C: M: 34.8±12.7 F: 32.4±11.0	P: (F:16, M:7) C: (F:878, M:355)
8	Coccaro EF [20]	2016	USA	Case control	ELISA	IgG	Not significant	P: 31.3±8.7 C: 33.7±8.1	P: (-) C: (F:46, M:64)
9	Flegr and Horáček a [15]	2017	Czech Republic	Cross sectional	CFT ELISA	IgG IgM	Not significant	M: 35.6±12.4 F: 32.9±12.3	P: (F:20, M:6) C: (F:1023, M:283)
10	Flegr and Horáček b [21]	2017	Czech Republic	Cross sectional			Not significant	M: 34.8±12.0 F: 30.5±10.9	P: (F:70, M:71) C: (F:2006, M:1203)
11	Afsharpaiman Sh [26]	2017	Iran	Case control	ELISA EIA	IgG IgM		P: 8.56 ± 2.5 C: 8.42 ± 1.9	P: (F:25, M:23) C: (F:25, M:23)
12	Akaltun I [25]	2018	Turkey	Case control	ELISA	IgG	Significant	P: 15.1 ± 3.9 C: 14.7 ± 2.8	P: (F:35, M:25) C: (F:31, M:29)

ELISA: enzyme-linked immunosorbent assay, IFA: indirect immunofluorescence assay, CFT: complement fixation test, EIA: enzyme immunoassay, IgG: Immunoglobulin G, IgM: Immunoglobulin M, P: Pateint, C: Control, F: Female, M: Male, N: Number

**Table 2:** Description of data extracted of the included studies in the systematic review and meta-analysis of the association between toxoplasmosis and obsessive compulsive disorder.

No	Reference	N	Case: OCD+ (n)	Control: OCD- (n)	OCD+ & T+ (n, %)	OCD- & T+ (n, %)	OR (95% CI)	P-value
1	Alvarado-Esquivel C [28]	181	1	180	0 (0%)	16 (8.9%)	3.32 (0.13–84.89)	0.91
2	Miman O [14]	142	42	100	20(47.62%)	19 (19%)	3.88 (1.77–8.50)	<0.01
3	Xiao Y [27]	2646	12	2634	3 (25%)	329 (12.5%)	2.34 (0.63–8.67)	
4	Cong W [29]	474	29	445	7 (24.14)	55 (12.36%)	2.26 (0.92–5.53)	0·068
5	Memik NÇ [16]	87	42	45	2 (4.8%)	4 (8.9%)	0.51 (0.09–2.96)	0.677
6	Zaki WM [24]	170	8	162	2 (25%)	24 (14.8%)	1.92 (0.37–10.06)	0.582
7	Flegr J [19]	1256	23	1233	3 (13.04%)	290 (23.52%)	0.49 (0.14–1.65)	0·126
8	Coccaro EF [20]	115	5	110	1 (20%)	10 (9.1%)	2.50 (0.25–24.58)	0.778
9	Flegr and Horáček a [15]	1332	26	1306	11 (42.31%)	355 (27.18%)	1.96 (0.89–4.32)	0.047
10	Flegr and Horáček b [21]	3350	141	3209	31 (22%)	516 (16.07%)	1.47 (0.98–2.22)	0.014
11	Afsharpaiman Sh [26]			48	1(2.08)	1(2.08)		
12	Akaltun I [25]	120	60	60	21 (35%)	6 (10%)	4.85 (1.79–13.13)	0.001

N and n: Number, CI: Confidence interval; OCD^+^: Individuals with obsessive compulsive disorder; OCD^-^: Individuals without obsessive compulsive disorder; OCD^+^ & T^+^: Individuals with obsessive compulsive disorder and *Toxoplasma* positive; OCD^-^ & T^+^: Individuals without obsessive compulsive disorder and *Toxoplasma* positive; OR: Odds ratio

Studies were published from 2006 to 2018. Accordingly, 9 out of the 12 studies had a case-control design, and 3 of them were cross-sectional studies (Table 1). One of the articles was not analyzed due to the unclear data about the exact number of patients with OCD [26]. The total number of participants involved in the 11 included studies in the meta-analysis was 9873, including 389 OCD patients and 9484 controls. Studies were conducted in Turkey [14,16,25], Czech Republic [15,19,21], China [27,29], USA [20], Mexico [28], Saudi Arabia [24], and Iran [26]. Anti-*Toxoplasma* antibodies (IgG and IgM) were determined using enzyme-linked immunosorbent assay [14–16,19,20,24–29], indirect immunofluorescence assay [14], complement fixation test [15,19], and enzyme immunoassays [26]. One of the studies did not address the method through which *Toxoplasma* is diagnosed [21].

**Fig 2:**
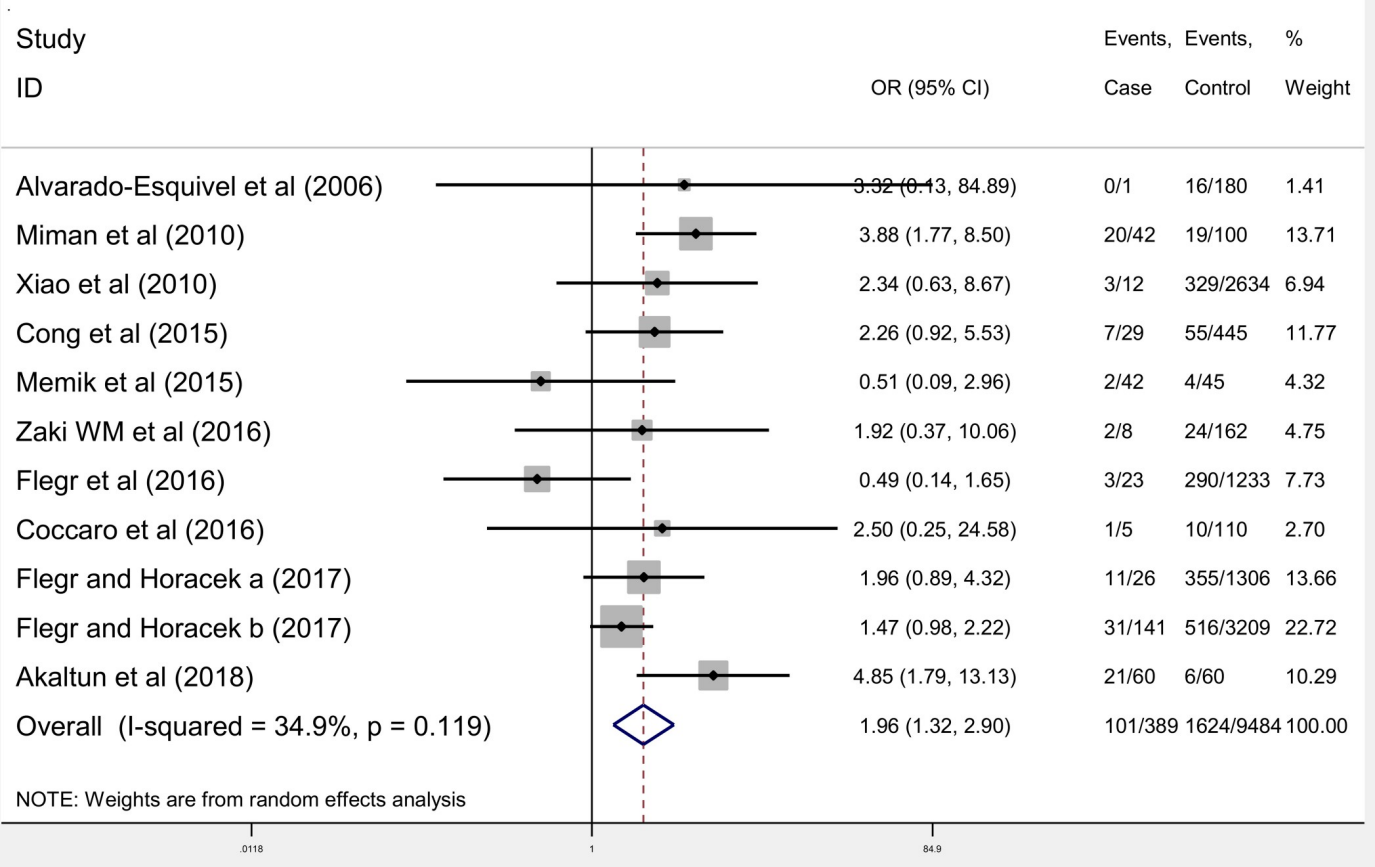
Meta-analysis of studies on the correlation between toxoplasmosis and OCD.

Meta-analysis results showed that the OR of the chance of toxoplasmosis in OCD patients compared to control groups was 1.96 (95% CI: 1.32–2.90) (Fig 2). The test of heterogeneity showed a moderate heterogeneity among the studies included in the meta-analysis (chi^2^ = 15.37, P = 0.119, I^2^ = 34.9%).

**Fig 3:**
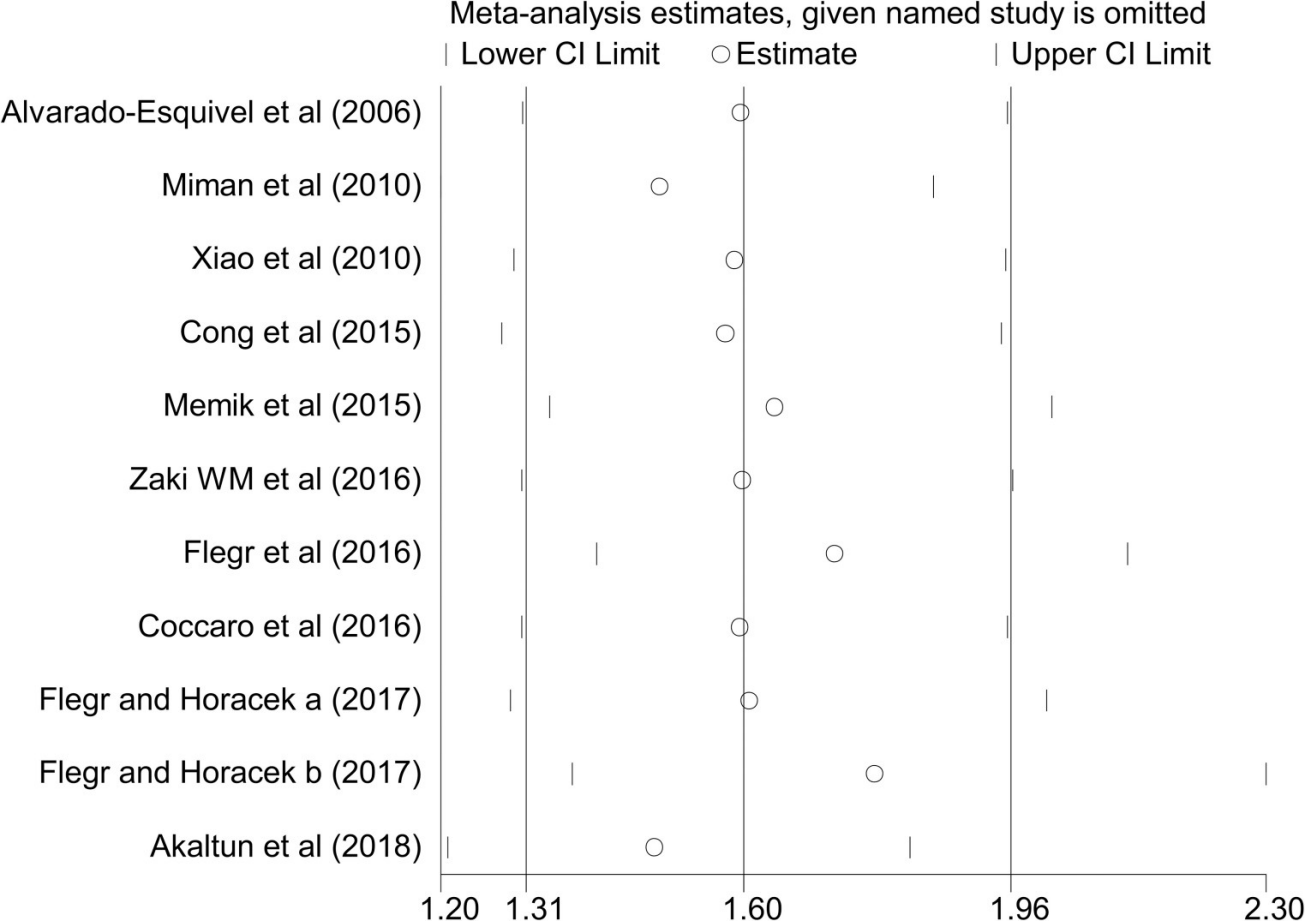
Sensitivity analysis for assessing the effect of each primary study on the total estimates.

Publication bias was assessed by Egger’s test and the results showed no publication bias (P = 0.540). Sensitivity analysis using the “one study removed at a time” technique demonstrated that the impact of each study on meta-analysis was not significant on the overall estimates (Fig 3).

## Discussion

Toxoplasmosis in the individuals leads to psychotic symptoms and changes in personality [3]. The *T*. *gondii* has a relationship with schizophrenia [30,31] and bipolar disorder [3] ]; however, its relationship with the OCD is understudied and there are few documented findings. The inconsistent results among the included studies in our meta-analysis demonstrate a discrepancy in the relationship between *T*. *gondii* and the chance of OCD. Therefore, we designed this systematic review and meta-analysis to assess the overall prevalence and ORs of this infection in the individuals with OCD compared to those in the control group.

A total of 12 articles on the prevalence of toxoplasmosis in OCD patients were included in the current paper. Although few studies were included in this meta-analysis, our findings indicated higher *T*. *gondii* seropositivity in the OCD patients compared to those in the control group with the OR of 1.96 (95% CI: 1.32–2.90). This agrees with the results of the ecological study by Flegr [32] showing a very strong correlation between incidence of toxoplasmosis and OCD-related burden in European (p = 0.02) and especially in non-European countries (p<0.0001). These results showed that there is a strong correlation between the prevalence of toxoplasmosis and OCD. The results of the current study 1.96 (95% CI: 1.32–2.90) differed from those of previous meta-analysis 3.4 (95% CI: 1.73–6.68) [6]. The previous meta-analysis was performed only on the basis of two studies in 2015 [6]. Since the current study investigates the updated evidence of the association between toxoplasmosis and OCD, it includes nine studies, which were not examined in the previous meta-analysis [15,16,19–21,24,25,28,29]. Moreover, a published study in 2006 was not included in the previous study [28] and this leads to discrepancies in the results of our study with the previous ones.

The included studies in our meta-analysis study were from three continents of Asia (Turkey: 3 studies, China: 2 studies, Iran: 1 study, Saudi Arabia: 1 study), Europe (Czech and Slovak Republics: 3 studies), and America (USA: 1 study, Mexico: 1 study). However, data gaps were identified for Africa, Australia, and many European countries where no data were available.

The status of the disease mainly depends on two quantities, the sensitivity and the specificity of the serological tests. However, all of the relevant studies have presented the prevalence of disease without mentioning tests sensitivity and specificity. Nevertheless, false positive and negative results can be significant because they do not show the prevalence of the infected people [33]. Variation in the sensitivity and specificity of enzyme-linked immunosorbent assay kits and the different cutoff values are effective factors on the prevalence of infection [34]. Different results of studies evaluated the relationship between various variables (including age, sex, education level, and history of blood transfusion) and the prevalence of toxoplasmosis reduced the ability to meta-analysis for these variables. In addition, the lack of evaluation of various associated factors in the eligible studies can be considered as basic gaps.

Identification of time evaluation is considered as an important variable for the temporal relationship between *T*. *gondii* exposure and disease onset. The evaluation of this variable helps to improve the precision of future studies describing the association between infectious agents and psychiatric disorders. However, none of the studies included in the current article considered this variable. Yolken et al. in 2017 [35] conducted a study for measured serological evidence of exposure to *T*. *gondii* in people. The results of the study indicated an increased odds of *T*. *gondii* exposure in people with a recent onset of psychosis (OR = 2.44).

Since the genetic characteristics of an individual can influence the forms of OCD family; therefore, there is a need to consider this issue in evaluating the relationship between OCD and *T*. *gondii*. However, only one study has addressed this important variable [26]. Rh phenotype is also an important variable that should be considered in various studies. Recent studies on women showed that Rh-positive women had lower levels of depression, obsession, and other psychiatric disorders. Although Rh-positive is an important variable, it has not been sufficiently addressed in previous studies [36]. The prevalence of *T*. *gondii* in patients with OCD was different among various studies. This difference in the prevalence of the included studies might be explained by the difference in the prevalence reported in the general population of each studied place. One of the reasons for the difference in the prevalence among the psychiatric and control populations might be due to the differences in the sanitary conditions among the groups. Indeed, most psychiatric inpatients belonged to a lower socio-economic level and had lower housing conditions than the control populations [28].

Some of the psychiatric disorders in humans are due to the ability of *T*. *gondii* to alter immune responses and neurotransmitters [37]. One of the important neurotransmitters is dopamine, which plays an essential role in the etiology of different neuropsychological diseases, such as major depression, schizophrenia, Parkinson’s disease, and Alzheimer’s disease [38]. Latent toxoplasmosis significantly affects dopaminergic and glutamatergic systems [39]. The higher chance of schizophrenia and OCD in the *T*. *gondii* infected individuals can be due to the increased dopaminergic activity [40]. Additionally, current studies have reported that brain cells infected with *Toxoplasma* contain high concentrations of dopamine [25]. The migration of *Toxoplasma* to the brain, formation of cysts, and changes in the production of neurotransmitters, such as dopamine can lead to the high rate of OCD prevalence in people with serum positive for *T*. *gondii* [25]. Treatment of two children with toxoplasmosis and OCD using anti-protozoan medications decreased *Toxoplasma* antibodies and completely cured OCD [41]. Furthermore, treatment in a 34-year-old woman with AIDS and neurotoxoplasmosis consuming antiprotozoal decreased OCD symptoms [42]. These findings supported a possible relationship between toxoplasmosis and OCD.

It has been suggested that changes in the hypothalamic-pituitary-adrenal gland axis, immune reactions [43], hormonal disorders caused by *Toxoplasma* infection [44], neuroimmune function and serotonin function disorder could lead to OCD [25]. Moreover, OCD could be due to a dysfunctionality of the front striatal loops, involved in frontal differentiation, as well as the lack of inhibition of automatic behavior [45,46]. Furthermore, some immune-mediated basal ganglia processes may be operating in OCD [41]. Denys et al. reported the observation of reduced TNF-alpha production and NK cell activity in patients with OCD [47]. Regardless these facts, it is possible that the OCD could be the cause rather than the effect of the *Toxoplasma* infection. It should be reminded, however, that OCD-induced behavioral changes such as fear of contamination, repeated washing of hands and social avoidance reduce rather than increase the chance of toxoplasmosis [15]. It is still possible that some unknown factor influences both the chance of toxoplasmosis and OCD. Therefore, further studies will be necessary to clarify the nature of the association between *T*. *gondii* and OCD.

### Limitations

One of the limitations of the included studies in the present research was that the individuals were invited to participate in some of these studies through snowball sampling technique using Facebook, fliers, and electronic media [15, 19, 21]. In this regard, the researcher(s) posted a Facebook announcement to invite people to take part in diverse psychological, ethological, and psychopathological experiments. However, the samples recruited in the mentioned studies cannot be representative of the general population since all people do not have access to Facebook. Moreover, the provided information were not based on the medical records; therefore, there were possibilities of wrong or at least obsolete data. To clarify, some patients may be infected with *Toxoplasma* after being tested for the presence of anti-*Toxoplasma* antibodies using serological methods. This could result in positively biased incidence rates of particular disorders. Accordingly, the obtained results cannot be generalized to the whole population. In one of these studies, the questionnaire contained many questions related to sexual behaviors and sexual preferences [21]. As a result, the participants were composed of those who were interested in these topics. Another limitation was that some studies were conducted only on children and adolescents, which made it difficult to generalize the findings to the society as a whole [25, 29].

There were also, some limitations in our research, including (1) few numbers of studies that investigated the relationship between *T*. *gondii* infection and OCD, (2) small sample size in the included studies, (3) reports with various quality, (4) available studies with no sufficient information on disease status/severity, (5) lack of the published articles in many parts of the world regarding the seroprevalence of toxoplasmosis among patients with OCD, (6) lack of the evaluation of various associated factors, such as familial history and Rh phenotype.

### Conclusions

Based on the currently available data, *T*. *gondii* infection was more frequent in OCD patients than the control group. The results of this study were indicative of a probability of positive association between the prevalence rate of toxoplasmosis and OCD. However, many questions remained to be answered in future studies. Therefore, further research should be performed to evaluate the reduction rate regarding the prevalence of OCD following the treatment of toxoplasmosis and the recognition of the physiopathological mechanisms involved in *T*. *gondii* infection in OCD. Also, it is highly desirable to obtain empirical data from other parts of the world.

## Supporting information

S1 Checklist STROBE statement-checklist. (DOCX)

S2 Checklist PRISMA 2009 checklist. (DOC)
